# Genome-wide characterization and analysis of bZIP transcription factor gene family related to abiotic stress in cassava

**DOI:** 10.1038/srep22783

**Published:** 2016-03-07

**Authors:** Wei Hu, Hubiao Yang, Yan Yan, Yunxie Wei, Weiwei Tie, Zehong Ding, Jiao Zuo, Ming Peng, Kaimian Li

**Affiliations:** 1 Key Laboratory of Biology and Genetic Resources of Tropical Crops, Institute of Tropical Bioscience and Biotechnology, Chinese Academy of Tropical Agricultural Sciences, Xueyuan Road 4, Haikou, Hainan, 571101, People’s Republic of China; 2 Tropical Crops Genetic Resources Institute, Chinese Academy of Tropic Agricultural Sciences, Danzhou, Hainan, 571737, People’s Republic of China

## Abstract

The basic leucine zipper (bZIP) transcription factor family plays crucial roles in various aspects of biological processes. Currently, no information is available regarding the bZIP family in the important tropical crop cassava. Herein, 77 *bZIP* genes were identified from cassava. Evolutionary analysis indicated that MebZIPs could be divided into 10 subfamilies, which was further supported by conserved motif and gene structure analyses. Global expression analysis suggested that *MebZIPs* showed similar or distinct expression patterns in different tissues between cultivated variety and wild subspecies. Transcriptome analysis of three cassava genotypes revealed that many *MebZIP* genes were activated by drought in the root of W14 subspecies, indicating the involvement of these genes in the strong resistance of cassava to drought. Expression analysis of selected *MebZIP* genes in response to osmotic, salt, cold, ABA, and H_2_O_2_ suggested that they might participate in distinct signaling pathways. Our systematic analysis of *MebZIPs* reveals constitutive, tissue-specific and abiotic stress-responsive candidate *MebZIP* genes for further functional characterization *in planta*, yields new insights into transcriptional regulation of *MebZIP* genes, and lays a foundation for understanding of bZIP-mediated abiotic stress response.

Due to their sessile nature, plants cannot move to avoid unfavorable conditions, thus they have to cope with various harsh environmental factors^1^. To survive in those stressors, plants developed various mechanisms for their protection, such as synthesis of functional proteins with diverse functions. Expression of functional proteins is largely controlled by specific transcription factors (TFs)^2^. Among TF families, the basic leucine zipper (bZIP) transcription factor family is one of the largest and most conserved families. bZIP TFs are named according to the conserved bZIP domain that is composed of 60–80 amino acids and contains two functional regions: a basic region and a leucine zipper. These two regions were linked by a hinge region^3^,^4^. The basic region is conserved, in which an invariant motif N-x7-R/K-x9 with about 18 amino acid residues is responsible for nuclear localization and DNA binding. The following leucine zipper motif that consists of several repeats of leucine or other hydrophobic amino acids is involved in recognition and dimerization of bZIPs^3^,^4^,^5^.

In plants, there is considerable evidence showing that bZIP TFs play crucial roles in various aspects of biological processes, including organ differentiation, embryogenesis, seed maturation, flower and vascular development^3^. Increasing evidences have also indicated that bZIP TFs take part in the regulation of plants’ response to biotic and abiotic stress^3^,^5^. Phytohormone abscisic acid (ABA) plays a central role in plants responding to abiotic stress through regulating the expression of numerous genes and related physiological process. In recent years, ABA perception and signal transduction have begun to clarify, which showed that RCAR/PYR/PYL ABA receptors, group A PP2Cs, and SnRK2s control the ABA signaling pathway in land plants^6^. SnRK2s can activate AREB/ABFs by phosphorylation in the ABA-dependent signaling network induced by abiotic stress^5^,^6^. AREB/ABFs, belonging to group A bZIPs, have been confirmed to modulate transcription of ABA-dependent genes that carry the ABRE *cis*-element, resulting in ABA response or environmental adaptation. In Arabidopsis, accumulated evidence has suggested the roles of group A bZIPs, such as, AtbZIP39, AtbZIP36, AtbZIP38, AtbZIP66, AtbZIP40, AtbZIP35 and AtbZIP37, in abiotic stress response or ABA signaling^5^,^6^. For example, ABF2/AREB1 is activated by phosphorylation of class 3 SnRK2s and overexpression of ABF2/AREB1 increased plants resistance to drought stress and sensitivity to ABA^6^,^7^,^8^. In rice, some bZIP family members, including OsbZIP23, OsbZIP46, OsbZIP71, and OsbZIP16, was also confirmed to positively regulate plants resistance to abiotic stress^9^. Together, these evidences indicate that bZIPs are crucial transcription factors involved in plants response to abiotic stress.

To date, numerous bZIP family members have been identified by genome-wide analyses in various species^4^,^10^,^11^,^12^. However, no evidence is available regarding the bZIP family in the important tropical crop cassava. Cassava is the third most important crop after rice and maize in Africa, Asia, and Latin America^13^. Due to its high starch production and limited input, cassava can provide source of dietary carbohydrate for over 600 million people worldwide, and is also considered as a producer of industrial starch and bioethanol^14^,^15^. Notably, cassava can effectively utilize light, heat and water resources; thus it shows resistance to drought and low-fertility environment^16^. However, the mechanisms by which cassava responds to abiotic stress are poorly understood. Thus, understanding of the molecular mechanisms underlying the resistance of cassava to abiotic stress may provide effective methods for genetic improvement of stress resistance for cassava and other crops. Previously, we get the high-quality sequencing data of cassava wild ancestor and cultivated varieties, thus providing an excellent opportunity for genome-wide analysis of cassava genes^17^. Due to the significance of bZIP TFs in various aspects of biological processes, especially for its crucial roles in abiotic stress response and ABA signaling, the bZIP family was chosen as a candidate for a systematic analysis in cassava.

## Results

### Identification and evolutionary analysis of bZIPs in cassava

To extensively identify cassava *bZIP* genes, we use BLAST and Hidden Markov Model searches to search the cassava genome database with bZIP sequences from Arabidopsis and rice as queries. After these programs, 77 putative bZIP members were characterized from cassava, and further conserved domain detection confirmed that all the identified bZIPs harbor the conserved bZIP domain that is the basic characteristics of bZIP family. The 77 predicted full-length cassava bZIP proteins varied from 129 (MebZIP69) to 766 (MebZIP1) amino acid residues and the relative molecular mass ranged from 15.0 (MebZIP69) to 83.2 (MebZIP1) kDa, with isoelectric points in the range of 5.23–10.18 (Supplementary Table S1).

**Figure 1:**
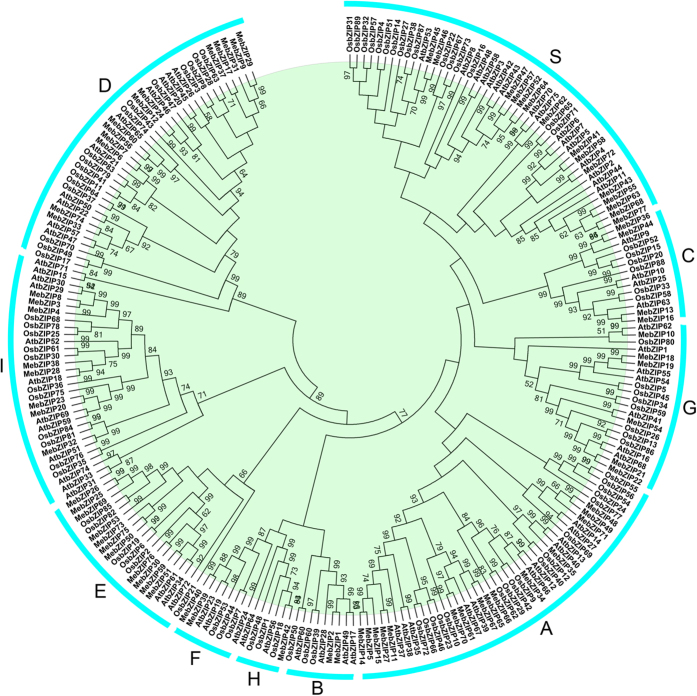
Evolutionary analysis of bZIP proteins from cassava, Arabidopsis, and rice. A total of 77 bZIPs from cassava, 72 bZIPs from Arabidopsis and 89 bZIPs from rice were used to creat the NJ tree with 1000 bootstrap. The bZIP proteins are grouped into 10 subgroups (A,B,C,D,E,F,G,H,I,S).

To characterize the evolutionary relationships between bZIPs from cassava and other known bZIPs from Arabidopsis and rice, an unrooted Neighbor-Joining tree was created (Fig. 1; Supplementary Table S2). The results showed that the 77 MebZIPs could be assigned to 10 subfamilies, together with their orthologous bZIPs from Arabidopsis and rice. Subfamily H only contains one MebZIP protein, whereas subfamilies A and S each have the maximum 15 MebZIP members, suggesting the existence of a diversified bZIP family in cassava with diverse functions. Generally, bZIPs from cassava have closer relationships with the bZIPs from Arabidopsis than that from rice, which is accord with the current understanding of plant evolutionary history. Evolutionary analysis also identified some closely related orthologous bZIPs between cassava and Arabidopsis, indicating that an ancestral set of *bZIP* genes existed prior to the divergence of cassava and Arabidopsis.

### Gene structure and conserved motifs of cassava *bZIPs*

**Figure 2:**
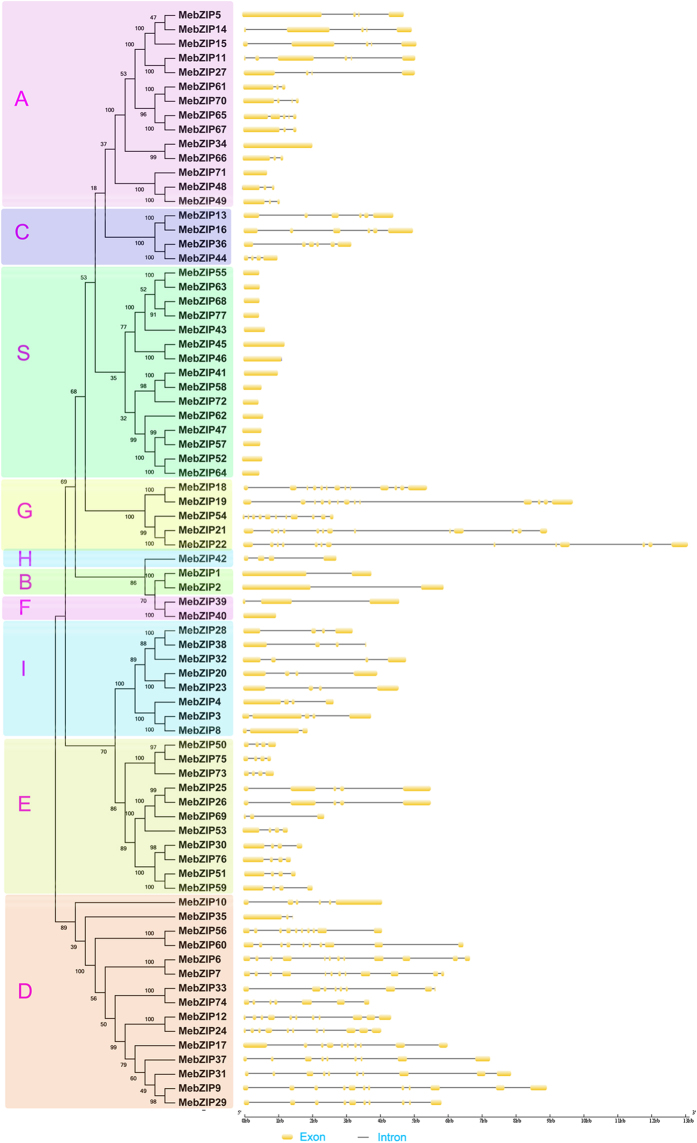
The exon-intron structure of *MebZIP* genes based on the evolutionary relationship. The NJ evolutionary tree was created with 1000 bootstrap based on the full length sequences of MebZIPs. Exon-intron analyses of *MebZIP* genes were carried out with GSDS. Lengths of exons and introns of each *MebZIP* gene were exhibited proportionally.

To better understand the structural features of MebZIP genes, intron/exon structure was detected based on their evolutionary relationships (Fig. 2). Gene structure analysis showed that the number of introns of MebZIP genes varied from 0 to 12. Subfamilies A, C, S, H, B, F, I, and E contained 0–5 introns, whereas subfamilies G and D had 5–12 introns, except for *MebZIP35*. Interestingly, no intron was detected in MebZIP genes of subfamily S. Generally, most of *MebZIP* genes in the same subfamilies showed similar exon-intron structure, which supports their close evolutionary relationship and the classification of subfamilies.

**Figure 3:**
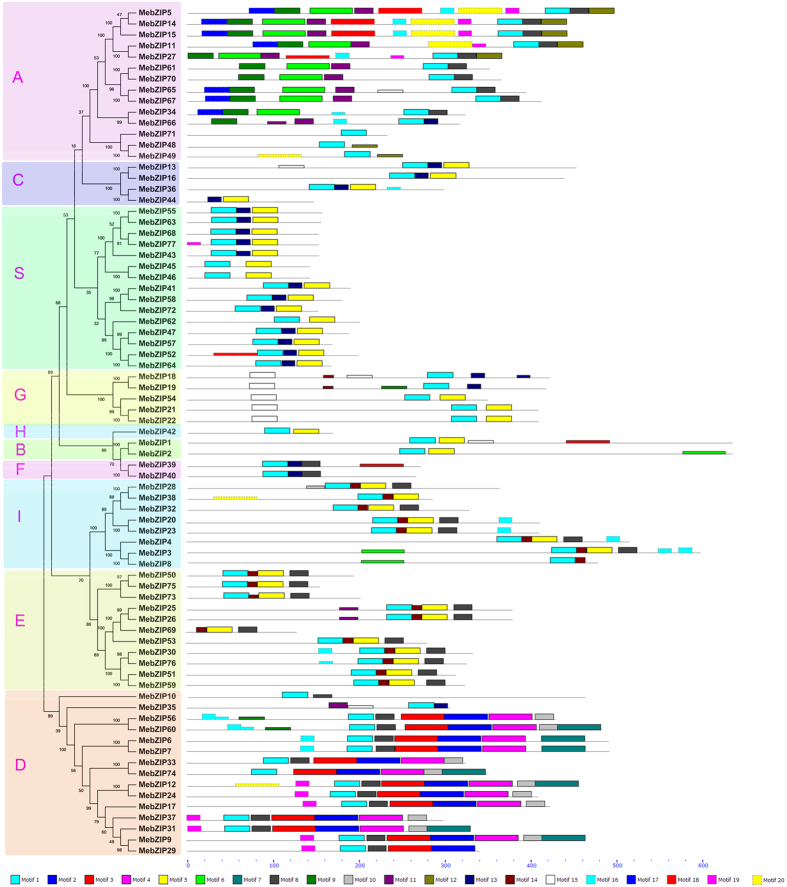
Conserved motifs of cassava bZIP proteins according to the evolutionary relationship. The conserved motifs in the MebZIP proteins were identified by MEME software. Grey lines represent the non-conserved sequences, and each motif is indicated by a colored box numbered at the bottom. The length of motifs in each protein was exhibited proportionally.

To investigate the structural diversity and functional prediction of MebZIPs, a total of 20 conserved motifs in the MebZIPs were captured by MEME software and subsequent annotation with InterPro (Supplementary Figure S1; Fig. 3; Supplementary Table S3). Motif 1 was annotated as basic-leucine zipper (bZIP) domain; motif 2 was annotated as transcription factor TGA like domain; and motif 15 was annotated as G-box binding, MFMR domain (Supplementary Table S3). Notably, all the MebZIPs, except for MebZIP44 and MebZIP69, had bZIP domain. MebZIP44 and MebZIP69 showed high identity with MebZIP36 and MebZIP26, respectively. It was observed that MebZIP44 and MebZIP69 exhibited C-terminal deficiency, which may result from the alternative splicing of MebZIP36 and MebZIP26, respectively. These two genes may not be functional bZIP transcription factors. Beside the bZIP domain, each subfamily of MebZIPs shares some common motifs. Most of bZIP members in subfamily A contain motifs 6, 8, 9, and 11; subfamily C shares motifs 5 and 13; subfamilies B, S, and H possess motif 5; subfamily G harbors motif 15; subfamily F shows motifs 8 and 13; motif 14 is present in subfamily I; motifs 5, 8, and 14 appeared in subfamily E; subfamily D have motifs 2 and 3. Additionally, some motifs represent the variable motifs across the bZIP gene family. For instance, motifs 9, 12, and 17 only appeared in subfamily A; and motifs 2, 4, 7, and 10 only appeared in subfamily D. These results indicated that bZIP members clustered in same subfamilies showed similar motif characteristic, suggesting functional similarities among members in the same subfamily.

### Expression profiles of *MebZIP* genes in different tissues of two cassava genotypes

**Figure 4:**
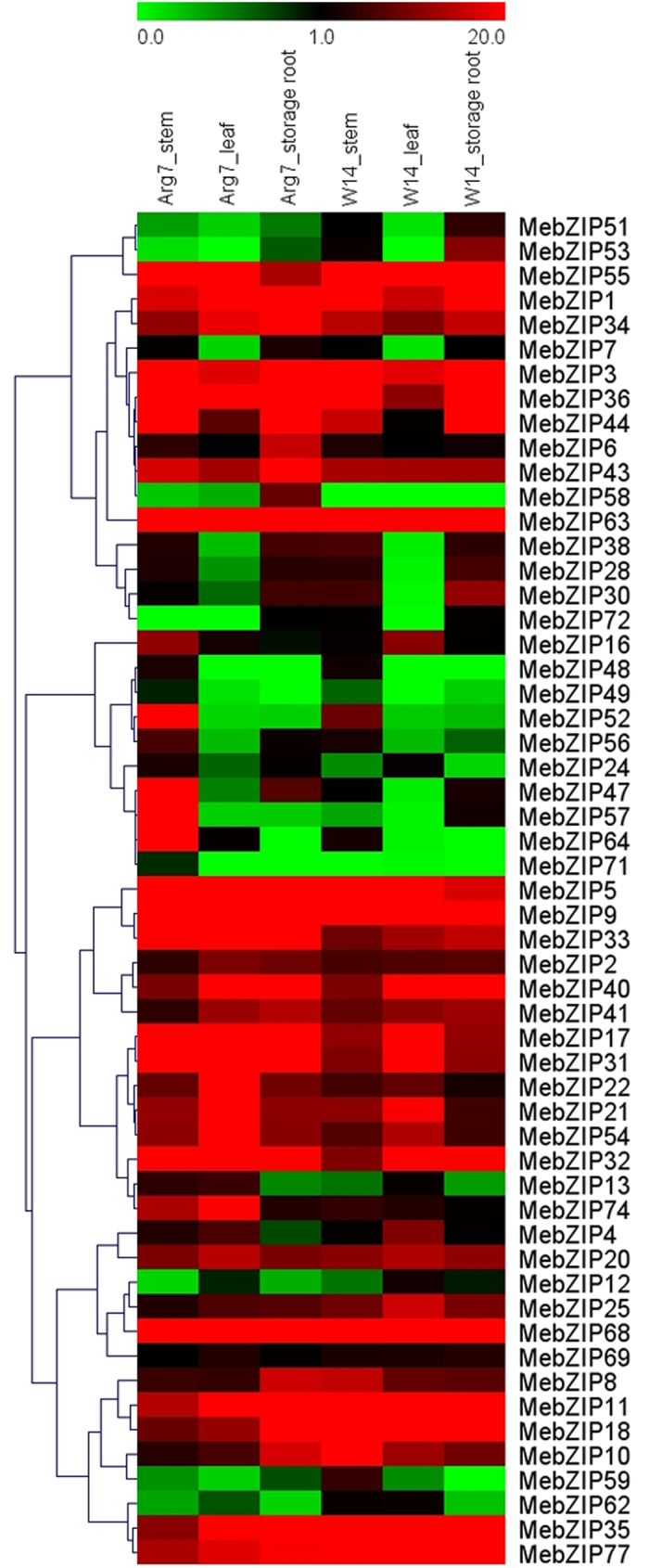
Expression profiles of *MebZIP* genes in different tissues of two cassava genotypes. FPKM value was used to create the heat map with clustering. The scale represents the relative signal intensity of FPKM values.

To seek insights into the roles of *MebZIP* genes in cassava development, total RNA was isolated from stems, leaves, and storage roots of cultivated varieties (Arg7) and wild subspecies (W14) for transcriptome analysis. Transcriptome analysis showed a transcript abundance of 55 *MebZIP* genes in different tissues, while the rest of the 22 *MebZIP* genes were not covered in the RNA-seq libraries (Fig. 4; Supplementary Table S4).

For Arg7 variety, all the 55 *MebZIP* genes (100%) showed transcripts in different tissues, in which 28 (50.9%), 26 (47.3%), and 27 (49.1%) genes exhibited high transcriptional abundance (value >10) in stem, leaf, and storage root tissues, respectively. Moreover, there were 21 *MebZIP* genes (*MebZIP-9*, *-5*, *-63*, *-36*, *-68*, *-3*, *-1*, *-11*, *-40*, *-43*, *-33*, *-17*, *-34*, *-31*, *-32*, *-35*, *-77*, *-55*, *-21*, *-54*, and *-20*) that had high expression levels (value >10) in all the tested tissues. Additionally, *MebZIP72* did not show transcripts in stem; *MebZIP-72*, *-53*, *-48*, *-71* did not show transcripts in leaf; and *MebZIP-64*, *-49*, *-48*, and *-71* did not show transcripts in storage root.

For W14 subspecies, all the 55 *MebZIP* genes, except for *MebZIP58*, had transcripts in different tissues, in which 23 (42.6%), 27 (50%), 25 (46.3%) genes had high transcriptional abundance (value >10) in stem, leaf, and storage root tissues, respectively. Moreover, 19 *MebZIP* genes (*MebZIP-68*, *-55*, *-63*, *-9*, *-3*, *-36*, *-1*, *-40*, *-18*, *-77*, *-35*, *-32*, *-11*, *-5*, *-34*, *-43*, *-17*, *-31*, and *-20*) showed high transcriptional levels (value > 10) in all the tested tissues. Additionally, *MebZIP-71* and *-58* did not show transcripts in stem; *MebZIP-53*, *-72*, *-49*, *-48*, and *-58* did not show transcripts in leaf; and *MebZIP-59*, *-64*, *-71*, *-48*, and *-58* did not show transcripts in storage root.

For comparing the expression profiles of *MebZIP* genes in different tissues between Arg7 and W14, 49 genes (89.1%) had transcripts accumulation in all tissues of Arg7, while 47 genes (85.5%) in W14, suggesting the constitutive expression patterns for these genes. On the contrary, the absence of detection might represent the distinct temporal or spatial expression patterns for the remaining *MebZIP* genes. Notably, 18 *MebZIP* genes (*MebZIP-9*, *-5*, *-63*, *-36*, *-68*, *-3*, *-1*, *-11*, *-40*, *-43*, *-17*, *-34*, *-31*, *-32*, *-35*, *-77*, *-55*, and *-20*) showed high transcript abundance (value >10) in all tested tissues of Arg7 and W14, indicating key roles for these genes in tissue development. Overall, *MebZIP* genes had similar expression profiles in different tissues of Arg7 and W14, indicating that most of *bZIP* genes played similar roles in tissue development of the two genotypes. However, there were some genes that exhibited differential expression profiles. *MebZIP-18*, *-10*, and *-8* had high transcript abundance (value >10) in stem of W14, whereas low in stem of Arg7. On the contrary, *MebZIP-33*, *-52*, *-54*, *-74*, *-64*, *-16*, *-47*, and *-57* had high transcriptional abundance (value >10) in stem of Arg7, while low in stem of W14. This phenomenon was also observed in leaf and storage root of Arg7 and W14. These findings indicated differential roles for these genes in tissue development in different genotypes.

### Expression profiles of *MebZIP* genes responding to drought stress in different cassava genotypes

**Figure 5:**
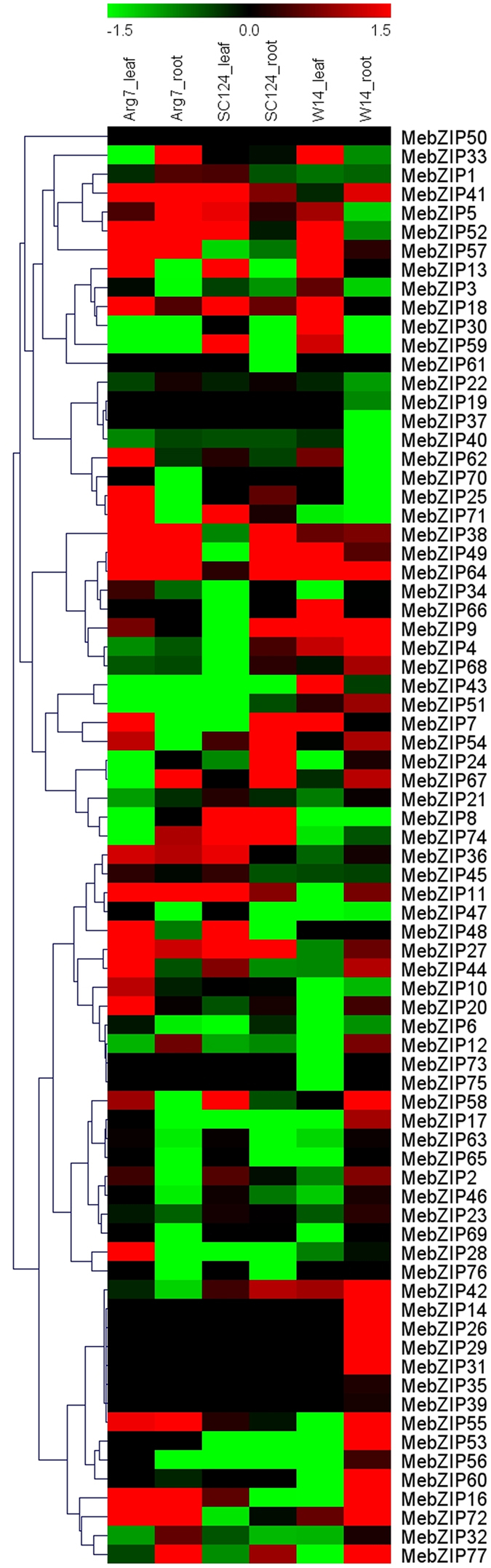
Expression profiles of *MebZIP* genes in leaves and roots of three cassava genotypes after drought treatment. Log2 based FPKM value was used to create the heat map with clustering. The scale represents the relative signal intensity of FPKM values.

To get some clues on the roles of *MebZIP* genes responding to drought stress, cassava seedlings of three genotypes were subjected to water withholding and then the leaves and roots tissues were sampled to extract RNA for subsequent RNA-seq analysis (Fig. 5; Supplementary Table S5). According to the transcriptome data, all the cassava *bZIP* genes, except for *MebZIP50*, showed the corresponding expression data. In Arg7 variety, 32 (42.1%) and 23 (30.3%) *MebZIP* genes were induced by drought in leaves and roots, respectively. In South China 124 (SC124) variety, 28 (36.8%) and 26 (34.2%) *MebZIP* genes showed upregulation after drought in leaves and roots, respectively. In W14 subspecies, 21 (27.6%) and 40 (52.6%) *MebZIP* genes were upregulated by drought in leaves and roots, respectively. These results suggest that the number of *MebZIP* genes upregulated by drought was greater in W14 than those in Arg7 and SC124, suggesting the comprehensive response of *bZIP* genes to drought at transcriptional levels in W14 subspecies. Based on the transcriptome data, we also found that the number of *bZIP* genes up-regulated by drought was significantly more in roots than those in leaves in W14, whereas less in Arg7 and SC124.

Generally, the expression patterns of *MebZIP* genes in response to drought were similar between Arg7 and SC124, which differs from W14. Seven genes (*MebZIP-33*, *-30*, *-43*, *-66*, *-4*, *-3*, and *-51*) showed induction in leaves of W14, whereas downregulation or no response in leaves of Arg7 and SC124 after drought treatment. Seventeen genes (*MebZIP-14*, *-26*, *-29*, *-60*, *-58*, *-53*, *-31*, *-44*, *-17*, *-51*, *-2*, *-56*, *-35*, *-46*, *-39*, *-63*, and *-21*) were upregulated in roots of W14, whereas downregulated or no response in roots of Arg7 and SC124 after drought treatment. This differential expression profile suggested that bZIP-mediated response underlying cassava tolerant to drought stress might be different between cultivated varieties and wild subspecies. In addition, some *bZIP* genes had close evolutionary relationships; however, they displayed different responses to drought stress, such as, *MebZIP-5/-14*, *MebZIP-65/-67*, *MebZIP-66/-34*, *MebZIP-48/-49*, *MebZIP-13/-16*, *MebZIP-36/-44*, *MebZIP-55/-63*, *MebZIP-68/-77*, *MebZIP-18/-19*, *MebZIP-1/-2*, and *MebZIP-39/-40*. Together, the expression profiles of *bZIP* genes responding to drought in cultivated varieties and wild subspecies will be benefit for further investigation of the mechanisms underlying strong drought resistance in cassava.

### Expression profiles of *MebZIP* genes upon exposure to various stress and related signaling

**Figure 6:**
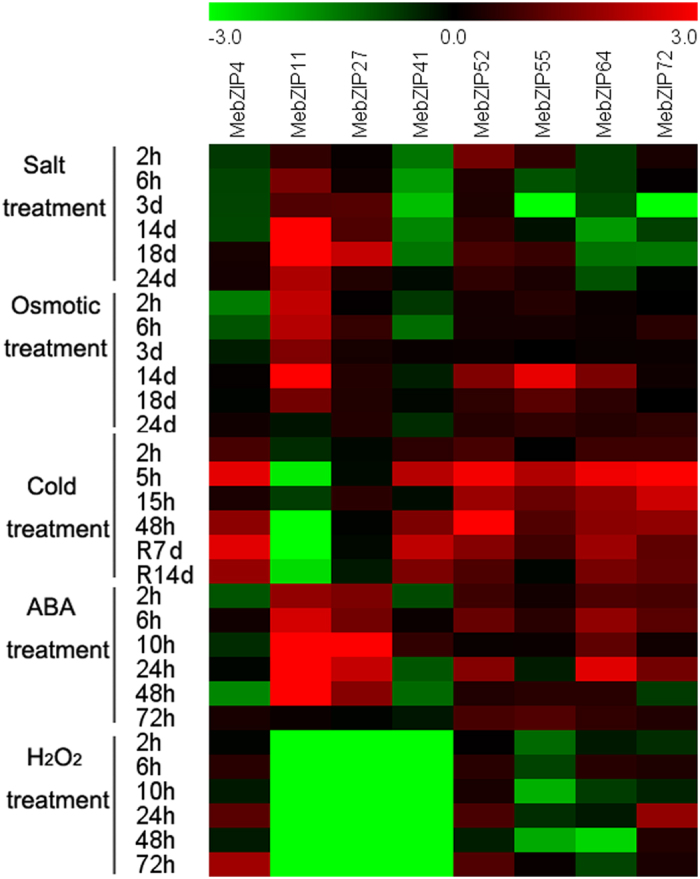
Expression profiles of *MebZIP* genes in leaves under various stimuli. Log2 based values from three replicates of quantitative real-time PCR data were used to create the heatmap. The scale represents the relative signal intensity values.

To examine the response of *MebZIP* genes to various environmental stresses and related signaling at transcriptional levels, the transcripts of *MebZIP* genes under these treatments were determined by quantitative real-time PCR. Eight *MebZIP* genes (*MebZIP-4*, *-11*, *-27*, *-41*, *-52*, *-55*, *-64*, and *-72*) induced by drought based on RNA-seq data in different cassava genotypes were chosen for further examination of their expression patterns after osmotic, salt, cold, ABA and H_2_O_2_ treatments (Fig. 6; Supplementary Table S6, S7). Under salt treatment, *MebZIP-11*, *-27*, and *-52* were upregulated, whereas *MebZIP-41* and *-64* were downregulated during all the treated time points. *MebZIP-4*, *-55*, and *-72* were repressed at several time points (Supplementary Figure S2). Under osmotic treatment, *MebZIP11* showed induction during 2 h–18d treatment. *MebZIP27* was induced after 6 h–24d treatment. *MebZIP-52*, *-55*, *-64*, and *-72* were upregulated at several time points. *MebZIP-4* and *-41* were repressed after 2–6 h treatment (Supplementary Figure S3). Under cold treatment following recovery, *MebZIP-4*, *-55*, *-64*, and *-72* transcripts increased during all the treated time points. *MebZIP-41* and *-52* showed upregulation at several time points. On the contrary, cold stress caused a seriously decrease in transcriptional levels of *MebZIP11*. *MebZIP27* did not show obvious trends after cold treatment (Supplementary Figure S4). Under ABA treatment, *MebZIP-11*, *-27*, *-52*, and *-64* were induced during all the treated time points. *MebZIP-55* and *-72* were upregulated at several time points. *MebZIP-4* and *-41* showed downregulation at several time points (Supplementary Figure S5). Under H_2_O_2_ treatment, *MebZIP-4* and *-52* were upregulated at several time points. *MebZIP55* transcripts decreased during 2–48 h treatment. *MebZIP-64* and *-72* did not show obvious trends after H_2_O_2_ treatment. Notably, *MebZIP-11*, *-27*, and *-41* were significantly repressed during all the treated time points (Supplementary Figure S6).

## Discussion

Cassava is the third most important crop after rice and maize in Africa, Asia, and Latin America. Research progress on cassava proceeded extremely slow compared to other important crops, such as rice, maize, wheat, and cotton. Cassava has the characteristic of strong resistances to drought and low-fertility environment. However, the mechanisms by which cassava responds to abiotic stress are poorly understood. The ABA signal pathway has been documented to play crucial roles in plants responding to abiotic stress, in which bZIP is important transcription factors for the ABA signaling pathway. Numerous studies have confirmed the roles of group A bZIPs (AREB/ABFs) in ABA response or environmental adaptation through regulating expression of ABA-dependent genes. However, the identity and function of cassava bZIPs have remained unknown.

Herein, we identified 77 *bZIP* family members from cassava. Previous studies have identified 75 *bZIP*s in Arabidopsis, 89 in rice, 92 in Sorghum, 64 in cucumber, 125 in maize, 55 in grapevine, 131 in soybean, and 96 in *Brachypodium distachyon*^4^,^10^,^11^. These data suggested that *bZIP* in cassava had expanded compared to that in Arabidopsis, cucumber, and grapevine, whereas had shrunk compared to that in rice, Sorghum, maize, soybean, and *Brachypodium distachyon*. Evolutionary analysis indicated that the cassava bZIPs could be clustered into 10 subfamilies, which is in line with previous evolutionary classification of bZIPs in Arabidopsis, grapevine and Sorghum^5^,^10^,^12^ (Fig. 1). This classification was further supported by gene structure analysis and conserved motif analysis. Gene structure analysis showed that the number of introns of *MebZIPs* varied from 0 to 12 (Fig. 2). In grapevine and *Brachypodium distachyon*, the number of introns varied from 0 to 10 and 0 to 13, respectively^4^,^10^. This suggested that there were similar gene structure diversity of *bZIP* genes in different species. We also observed that the number of introns was significantly more in subfamilies G and D than in subfamilies A, C, S, H, B, F, I, and E. According to a previous report^18^, the rate of intron loss is faster than the rate of intron gain after segmental duplication in rice. Thus, it can be concluded that subfamilies G and D might contain the original genes, from which those in other clusters were derived. Additionally, all the 15 *bZIP* members in subfamily S were intronless. This phenomenon was also found in other species, such as grapevine and *Brachypodium distachyon*^4^,^10^. Conserved motif analysis indicated that almost all the MebZIPs contained typical bZIP domain. Additioanlly, each subfamily had some common motifs and some subfamilies also contained the special motifs (Fig. 3). These features in conserved motifs of bZIPs were also observed in grapevine^10^. Generally, most of *MebZIP* genes in the same subfamilies showed similar gene structure and conserved motifs, which supports their close evolutionary relationship and the classification of subfamilies.

Although cassava has robust resistance to drought stress, the mechanisms by which cassava responds to abiotic stress are poorly understood. Genome-wide expression profiles have indicated that numerous *bZIP* genes showed transcriptional response to drought/osmotic stress^4^,^10^,^11^,^12^. Further biochemical and genetic evidences revealed that group A *bZIPs* played central roles in plants responding to abiotic stress and ABA signal in various species^5^,^19^,^20^,^21^,^22^. In this study, we found that many *MebZIP* genes could transcriptionally respond to drought stress in different genotypes, suggesting possible function of these genes in responsive to drought stress in cassava (Fig. 5). Further, we observed that the number of *MebZIP* genes upregulated by drought was greater in W14 than those in Arg7 and SC124, suggesting the comprehensive response of *bZIP* genes to drought at transcriptional levels in W14 subspecies (Fig. 5). W14 was reported to have strong resistance to drought stress^17^. After withholding water for 12 days, W14 showed less cured and wilted leaves relative to SC124 and Arg7, indicating that W14 was more tolerant to drought than SC124 and Arg7 (Supplementary Figure S7). Numerous evidences have confirmed that *bZIPs* play a positive role in response to drought or osmotic stress in plants^9^,^21^. Consequently, we speculated that the high ratio of *MebZIP* genes upregulated by drought in W14 might contribute to its robust drought resistance. Finally, we noted that the number of *bZIP* genes up-regulated by drought was significantly more in roots than those in leaves in W14, whereas less in Arg7 and SC124 (Fig. 5). Cassava can penetrate into deep soil layers and absorb water stored in soil because of its deep root system^23^. Therefore, the high ratio of drought induced *MebZIP* genes in roots indicates that *MebZIP* genes might function on water uptake from soil by roots, thus maintaining robust drought resistance of W14. Collectively, these findings suggested that *MebZIP* genes might contribute to the robust resistance of cassava to drought stress and provided a foundation for further research on the *bZIP*-mediated drought response in cassava.

To date, accumulated evidences have revealed the involvement of *bZIP* genes in abiotic stresses and related signal transduction pathways. However, no information was available for the response of *bZIP* genes to these stimuli in cassava. In this study, eight *MebZIP* genes (*MebZIP-4*, *-11*, *-27*, *-41*, *-52*, *-55*, *-64*, and *-72*) upregulated by drought based on RNA-seq data in different cassava genotypes were chosen for further examination of their transcriptional response various treatments (Fig. 6; Supplementary Table S6, S7).

Under salt treatment, *MebZIP-11*, *-27*, and *-52* showed upregulation, whereas *MebZIP-41*, *-64*, *-4*, *-55*, and *-72* exhibited downregulation at several time points (Supplementary Figure S2). There was some evidences showing that some *bZIP* genes positively regulate salt resistance, such as, *ABF2*, *AtbZIP17*, *OsABI5*, *OsbZIP23*, *OsbZIP71*, *GmbZIP44*, *GmbZIP62*, and *GmbZIP78*^9^,^19^,^24^,^25^,^26^. Conversely, *ABF3* and *ABF4* overexpressing Arabidopsis plants showed salt-hypersensitive phenotype, indicating their negative effect on salt response^27^. These data suggested that *bZIP* family genes might be positively or negatively involved in salt stress response.

Biochemical and genetic evidences have suggested that *bZIP* genes, such as, *ABF2*, *ABF3*, *ABF4*, *OsbZIP23*, *OsbZIP46*, *OsbZIP71*, and *OsbZIP16* could positively regulate plants resistance to osmotic/drought stress, indicating the crucial role of *bZIP* genes in osmotic/drought stress response^9^,^21^. We found that most of the selected *MebZIP* genes were upregulated after osmotic treatment in cassava (Supplementary Figure S3). In *Brachypodium distachyon*, 16 of 96 *bZIP* genes were upregulated; conversely, 28 of 96 genes were downregulated after 6 h osmotic treatment^4^. In sorghum, 6 of 16 *bZIP* genes showed induction, whereas only one exhibited downregulation after osmotic treatment^12^. Together, these results suggest the important roles of *bZIP* genes responding to osmotic/drought stress.

Cold stress does great harm to the growth and development of cassava, thus leading to decrease of cassava production. However, little is known for the mechanism underlying cassava response to cold stress. In the present study, we found that all the tested *bZIP* genes, except for *MebZIP11*, showed upregulation following cold treatment (Supplementary Figure S4). In *Brachypodium distachyon*, 12 of 96 *bZIP* genes were upgulated; conversely, 28 of 96 genes were downregulated after 6 h cold treatment^4^. In chinese cabbage, transcripts of 36 of 136 *bZIP* genes increased, while 17 of 136 genes decreased after cold treatment^28^. These results will be benefit for future researches on the function of *bZIP* genes and mechanisms underlying cold response.

The phytohormone ABA plays a crucial role in plants responding to abiotic stress, including drought, osmotic, salt and cold stresses. Accumulated evidence has suggested that some *bZIP* members are crucial components of ABA signaling pathway. In Arabidopsis, *ABF2*, *ABF3*, and *ABF4* were significantly upregulated by ABA and abiotic stress treatments. Further functional analyses showed that these genes were involved in the regulation of ABA-mediated processes, such as root elongation and seed germination^21^. To study the response of *bZIP* genes to ABA signaling, we examined the transcriptional levels of 8 *MebZIP* genes following ABA treatment. The results found that 6 genes were upregulated, and 2 genes were downregulated after ABA treatment, indicating the comprehensive response of *bZIP* genes to ABA in cassava (Supplementary Figure S5).

H_2_O_2_, as an important signal molecule, is induced by environmental and developmental stimuli^29^. To determine whether cassava *bZIP* genes are involved in H_2_O_2_ signaling pathway, we examined the expression changes of 8 *MebZIP* genes after H_2_O_2_ treatment. The results showed that *MebZIP-4*, and *-52* were upregulated at several time points, whereas *MebZIP-55*, *-11*, *-27*, and *-41* were significantly repressed after H_2_O_2_ treatment (Supplementary Figure S6). This is consistent with the expression patterns of *bZIPs* in response to H_2_O_2_ in *Brachypodium distachyon*, in which 6 genes were induced, and 28 genes were repressed^4^. These results indicated that H_2_O_2_ might mainly cause downregulation of *bZIP* genes.

In conclusion, we identified 77 *bZIP* genes from cassava and built their basic classification and evolutionary relationship using evolutionary, conserved protein motif, and gene structure analyses, which will supply abundant information for functional characterization of *bZIP* genes. The expression profiles of *MebZIPs* in distinct tissues of two cassava genotypes indicated that *bZIP* members showed similar or differential expression patterns between Arg7 and W14, thus assisting in understanding the molecular basis for tissue development and function. Transcriptome analysis of three cassava genotypes responding to drought stress revealed that more *MebZIP* genes were activated in the root of W14 subspecies compared with Arg and SC124, which might contribute to its robust resistance to drought. Expression analysis of *MebZIP* genes under various treatments showed the comprehensive response of cassava *bZIP* genes to salt, osmotic, cold, ABA, and H_2_O_2_, indicating that they might represent convergence points of different signaling pathways. These data will lay a solid foundation for future research on the functional characterization of *bZIP* genes and *bZIP*-mediated signal transduction pathways, thereby advancing our understanding of the molecular basis of genetic enhancements to the cassava.

## Methods

### Plant materials and treatments

The characteristics of cassava genotypes W14, SC124 and Arg7 have been described in previous studies^30^,^31^. Segments cut from cassava stems were inserted into pots filled with soil and vermiculite (1:1) where they were regularly watered. The plants were grown from April to July 2013 during which time the temperature in the glass house ranged from 20 to 35 °C. To analyze transcripts of cassava *bZIP* genes in different tissues, stems (90-days-old), leaves (90-days-old) and storage roots (150-days-old) were collected from Arg7 and W14 under standard conditions. To examine the transcriptional changes of cassava *bZIP* genes to drought stress, leaf samples (90-days-old) were collected from and W14, Arg7 and SC124 under standard conditions or drought treatments for 12 days. For various stimuli, 60-days-old seedlings of Arg7 were subjected to 300 mM NaCl for 14 d, 200 mM mannitol for 14 d, 100 μM abscisic acid (ABA) for 24 h, 10% H_2_O_2_ for 24 h, and low temperature (4 °C) for 48 h, respectively.

### Identification and evolutionary analyses

The whole bZIP protein sequence of cassava, *Arabidopsis* and rice was obtained from the Phytozome (http://www.phytozome.net/cassava.php), UniPort (http://www.uniprot.org/) and RGAP (http://rice.plantbiology.msu.edu/) databases, respectively^32^,^33^,^34^. To identify the cassava *bZIP* family genes, the local Hidden Markov Model-based searches (http://hmmer.wustl.edu/) was firstly built from known bZIPs to search the cassava genome database^35^; subsequently, BLAST searches were performed to check the predicted bZIPs in cassava database with all the *Arabidopsis* and rice bZIPs as queries. Finally, all candidate protein sequences were further examined by the CDD (http://www.ncbi.nlm.nih.gov/cdd/) and PFAM (http://pfam.sanger.ac.uk/) databases^36^,^37^. Then, multiple sequence alignments were applied to confirm the conserved domains of predicted MebZIP proteins. Additionally, sequence alignments of the full-length bZIP proteins from cassava, *Arabidopsis* and rice were performed by Clustal X 2.0. The bootstrap neighbor-joining evolutionary tree was created by MEGA 5.0 software with 1000 bootstrap replicates based on the sequence alignments^38^,^39^.

### Protein properties and sequence analyses

The ExPASy proteomics server (http://expasy.org/) was employed to detect the molecular weight and isoelectric points of predicted MebZIP proteins^40^. Using the MEME program (http://meme.nbcr.net/meme/cgi-bin/meme.cgi), the conserved motifs in full-length cassava bZIP proteins were identified with the following parameters: maximum number of motifs was 20 and the optimum width of motifs was set between 10 and 50^41^. Subsequently, all identified motifs were annotated with the help of InterProScan (http://www.ebi.ac.uk/Tools/pfa/iprscan/)^42^. The gene structure display server program (http://gsds.cbi.pku.edu.cn/) was employed to identify gene structures of cassava *bZIPs*^43^.

### Transcriptome analysis

Leaves and storage roots of Arg7 and W14 under standard conditions, and leaves and roots of Arg7, SC124 and W14 under normal conditions and 12-days drought treatment were collected to extract total RNA using plant RNeasy extraction kit (TIANGEN, Beijing, China) for transcriptome analysis. Three μg total RNA of each sample were used to construct the cDNA libraries following the Illumina instructions, and subsequently sequenced by Illumina GAII. Data preprocessing and analyses were performed according to our previous study^30^. The transcriptiomic data was submitted to NCBI and the accession number was listed in Supplementary Table S8.

### Quantitative real-time PCR analysis

Transcriptional changes of *MebZIP* genes responding to various stimuli, including osmotic, salt, cold, ABA and H_2_O_2_ were determined by quantitative real-time PCR analysis on Stratagene Mx3000P Real-Time PCR system using SYBR^®^ Premix Ex Taq™ (TaKaRa, Japan). The PCR amplification conditions used for all reactions were implemented as follows: 10 min at 95 °C, and followed by 40 cycles of 10 s at 95 °C, 15 s at 50 °C and 30 s at 72 °C. The relative expression levels of the target genes were calculated by the 2^–ΔΔCt^ method^44^. Reaction specificities for each primer pairs was tested using quantitative real-time PCR melting curve analysis, agarose gel electrophoresis and sequencing PCR products (Supplementary Table S9). β-tubulin gene (TUB) and elongation factors 1α gene (EF1) were employed as internal references to normalize the transcriptional levels of target genes^45^. Each treated sample contained a corresponding regularly-watered control and each sample had three independent biological replications. The treated and control plants at each time point were sampled for expression analysis. The relative expression levels of *MebZIP* genes in each treated time point were compared with that in each time point at normal conditions.

## Additional Information

**How to cite this article**: Hu, W. *et al.* Genome-wide characterization and analysis of bZIP transcription factor gene family related to abiotic stress in cassava. *Sci. Rep.* **6**, 22783; doi: 10.1038/srep22783 (2016).

## Supplementary Material

Supplementary Dataset 1

Supplementary Dataset 2
