# Supplementary material for: Genome-wide characterization and analysis of bZIP transcription factor gene family related to abiotic stress in cassava
Source: Sci Rep. 2016 Mar 7;6:22783. doi: 10.1038/srep22783 (PMC4780028; doi:10.1038/srep22783)
Supplement: Supplementary Dataset 1 [file srep22783-s1.doc]

**Supplementary information for following article**

**Genome-wide characterization and analysis of bZIP transcription**

**factor gene family related to abiotic stress in cassava**

Wei Hu^1#^*, Hubiao Yang^2#^, Yan Yan^1^, Yunxie Wei^1^, Weiwei Tie^1^, Zehong Ding^1^, Ming Peng^1^*, Kaimian Li^1^*

^1^Key Laboratory of Biology and Genetic Resources of Tropical Crops, Institute of Tropical Bioscience and Biotechnology, Chinese Academy of Tropical Agricultural Sciences, Xueyuan Road 4, Haikou, Hainan, 571101, People’s Republic of China

^2^Tropical Crops Genetic Resources Institute, Chinese Academy of Tropic Agricultural Sciences, Danzhou, Hainan, 571737, People’s Republic of China

*Corresponding author: Wei Hu ([huwei2010916@126.com](mailto:huwei2010916@126.com)); Ming Peng ([pengming@itbb.org.cn](mailto:pengming@itbb.org.cn)); Kaimian Li (likaimian@itbb.org.cn)

^#^ These authors contributed equally to this work.


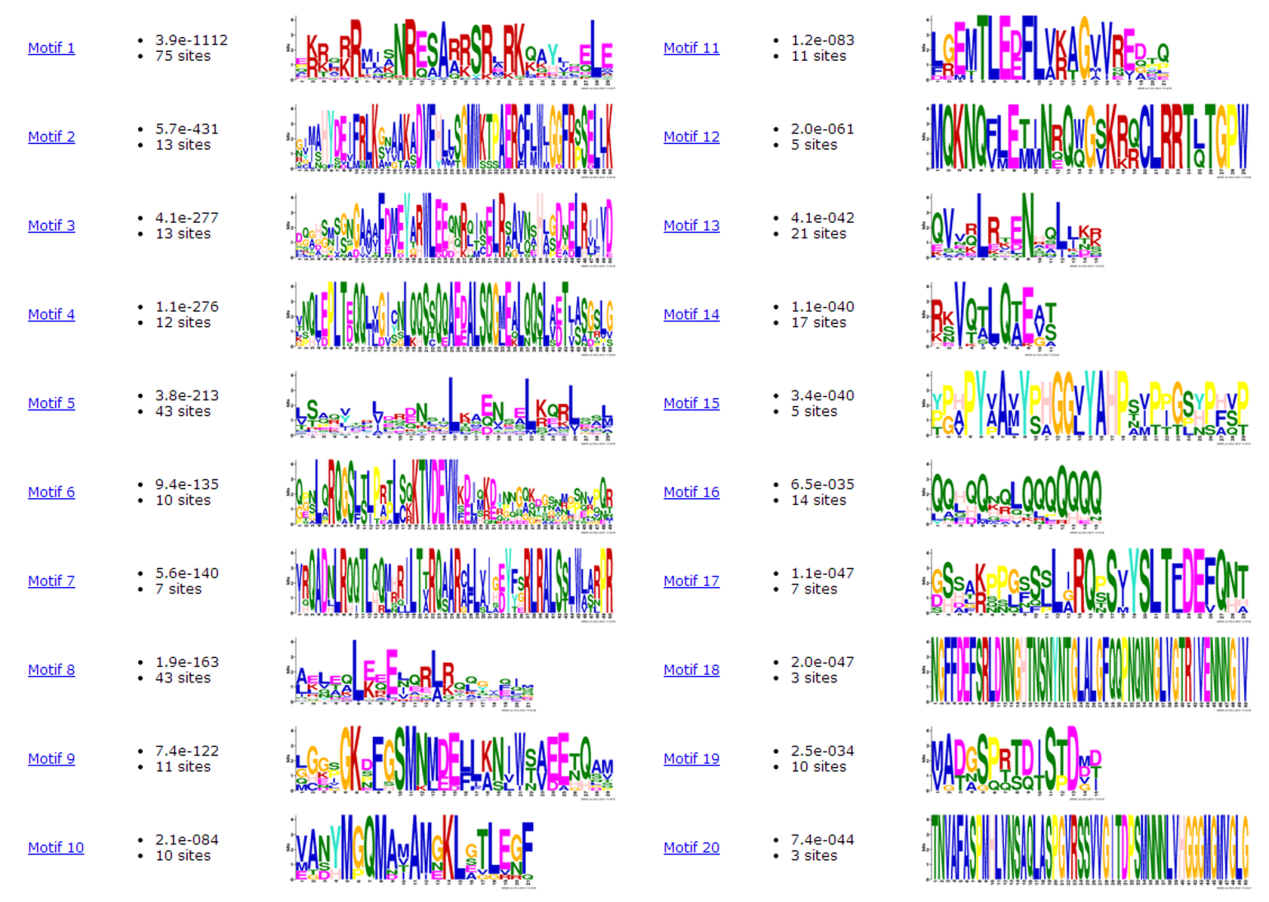


**Supplementary Figure S1. Sequence logos for conserved motifs identified in MebZIPs by MEME analysis.**


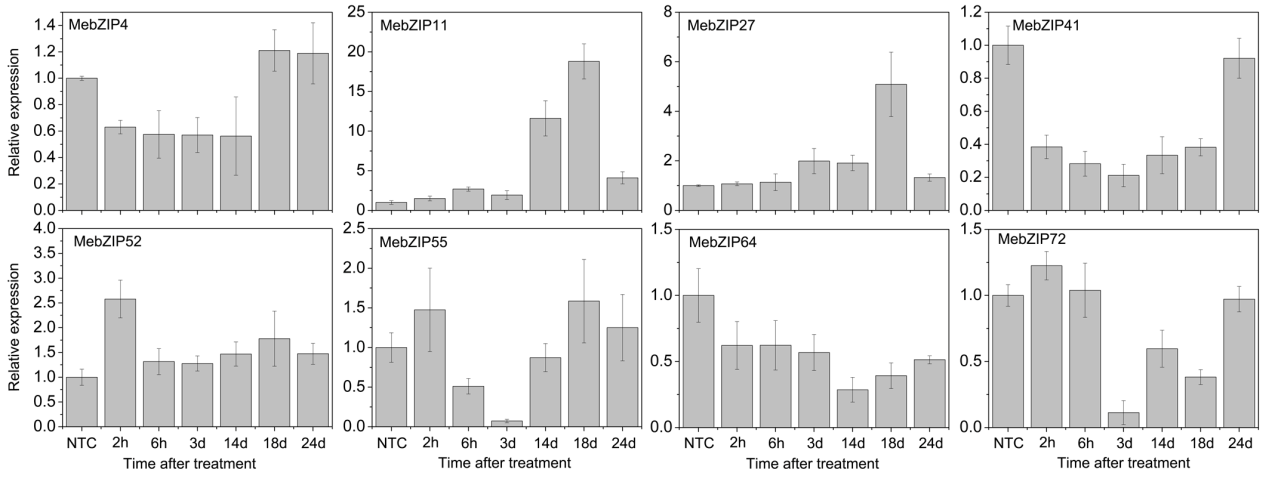


**Supplementary Figure S2. Expression profiles of *MebZIP* genes in leaves under salt stress.** The relative expression levels of *MebZIP* genes in each treated time point were compared with that in each time point at normal conditions. NTC (no treatment control) at each time point was normalized as “1”. Data are means ± SD calculated from three biological replicates.


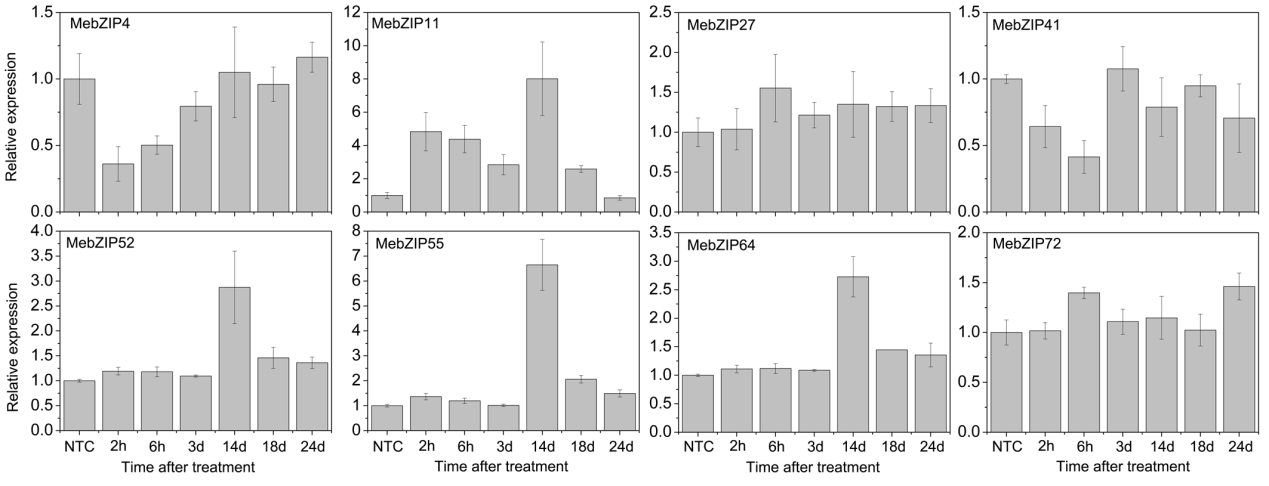


**Supplementary Figure S3. Expression profiles of *MebZIP* genes in leaves under osmotic stress.** The relative expression levels of *MebZIP* genes in each treated time point were compared with that in each time point at normal conditions. NTC (no treatment control) at each time point was normalized as “1”. Data are means ± SD calculated from three biological replicates.


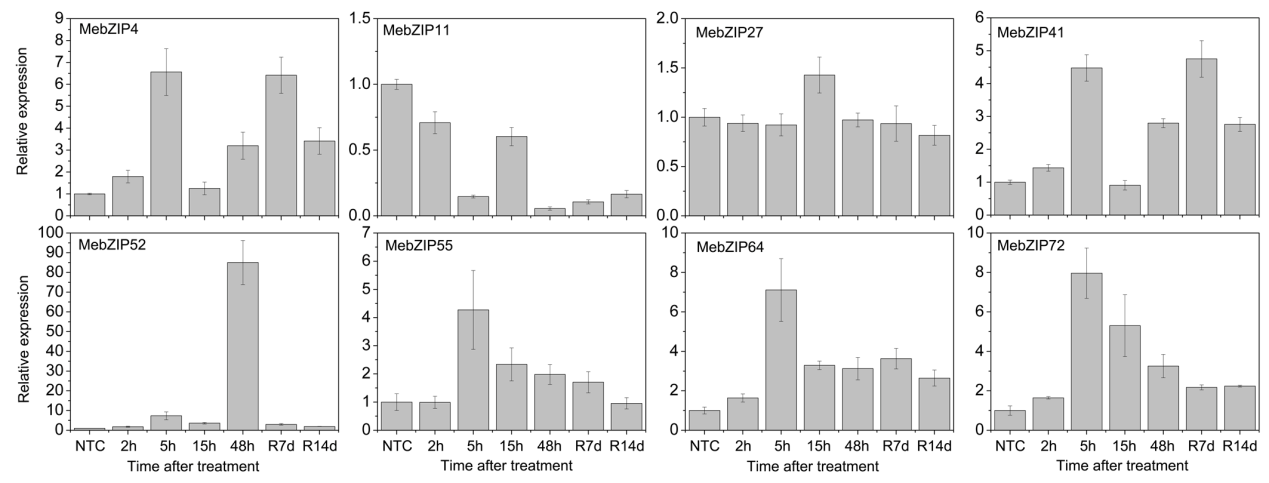


**Supplementary Figure S4. Expression profiles of *MebZIP* genes in leaves under cold stress.** The relative expression levels of *MebZIP* genes in each treated time point were compared with that in each time point at normal conditions. NTC (no treatment control) at each time point was normalized as “1”. Data are means ± SD calculated from three biological replicates.


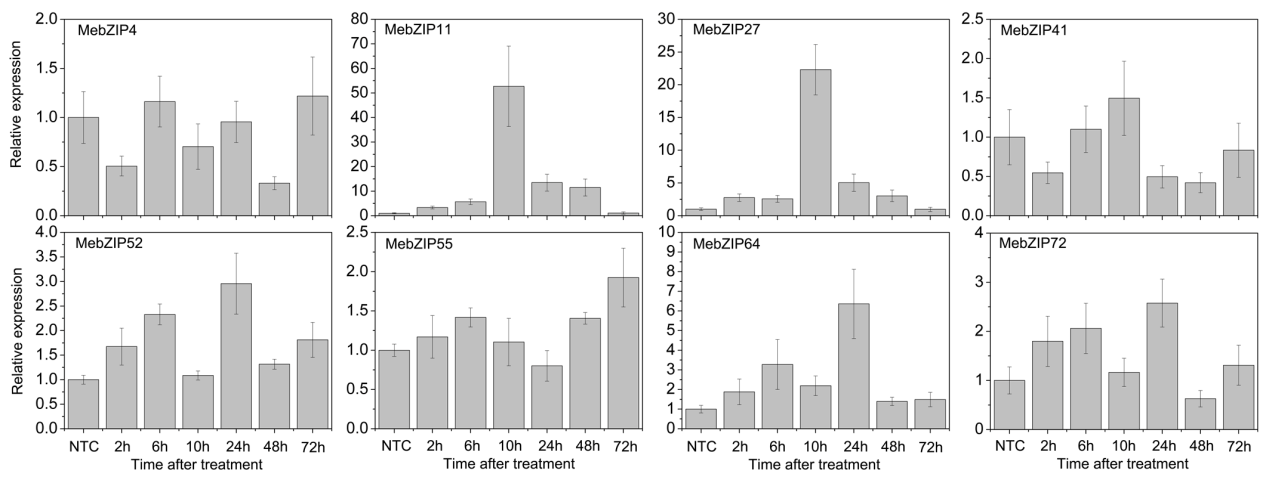


**Supplementary Figure S5. Expression profiles of *MebZIP* genes in leaves under ABA treatment.** The relative expression levels of *MebZIP* genes in each treated time point were compared with that in each time point at normal conditions. NTC (no treatment control) at each time point was normalized as “1”. Data are means ± SD calculated from three biological replicates.


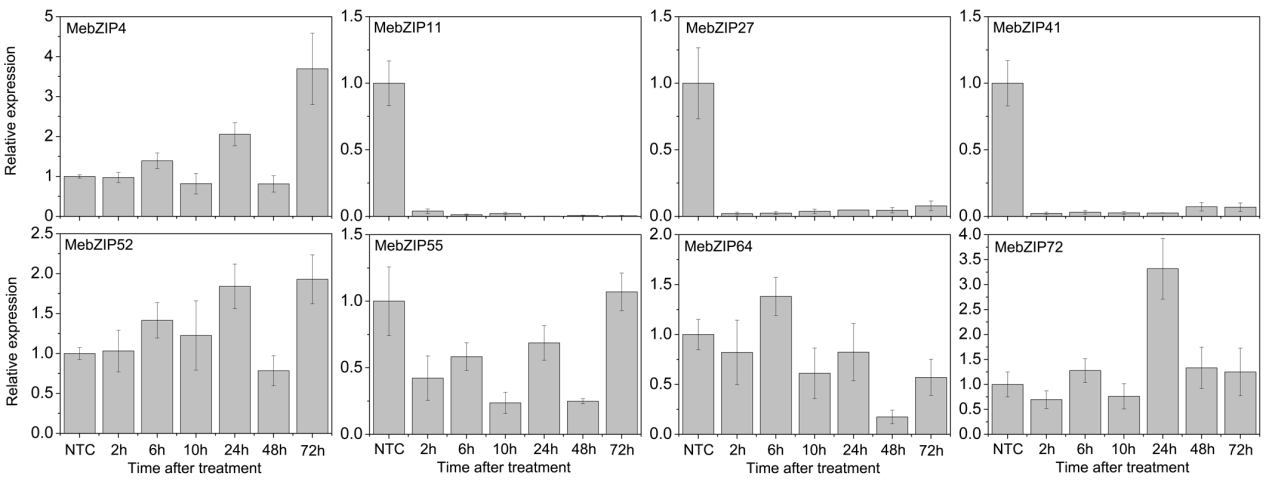


**Supplementary Figure S6. Expression profiles of *MebZIP* genes in leaves under H_2_O_2_ treatment.** The relative expression levels of *MebZIP* genes in each treated time point were compared with that in each time point at normal conditions. NTC (no treatment control) at each time point was normalized as “1”. Data are means ± SD calculated from three biological replicates.


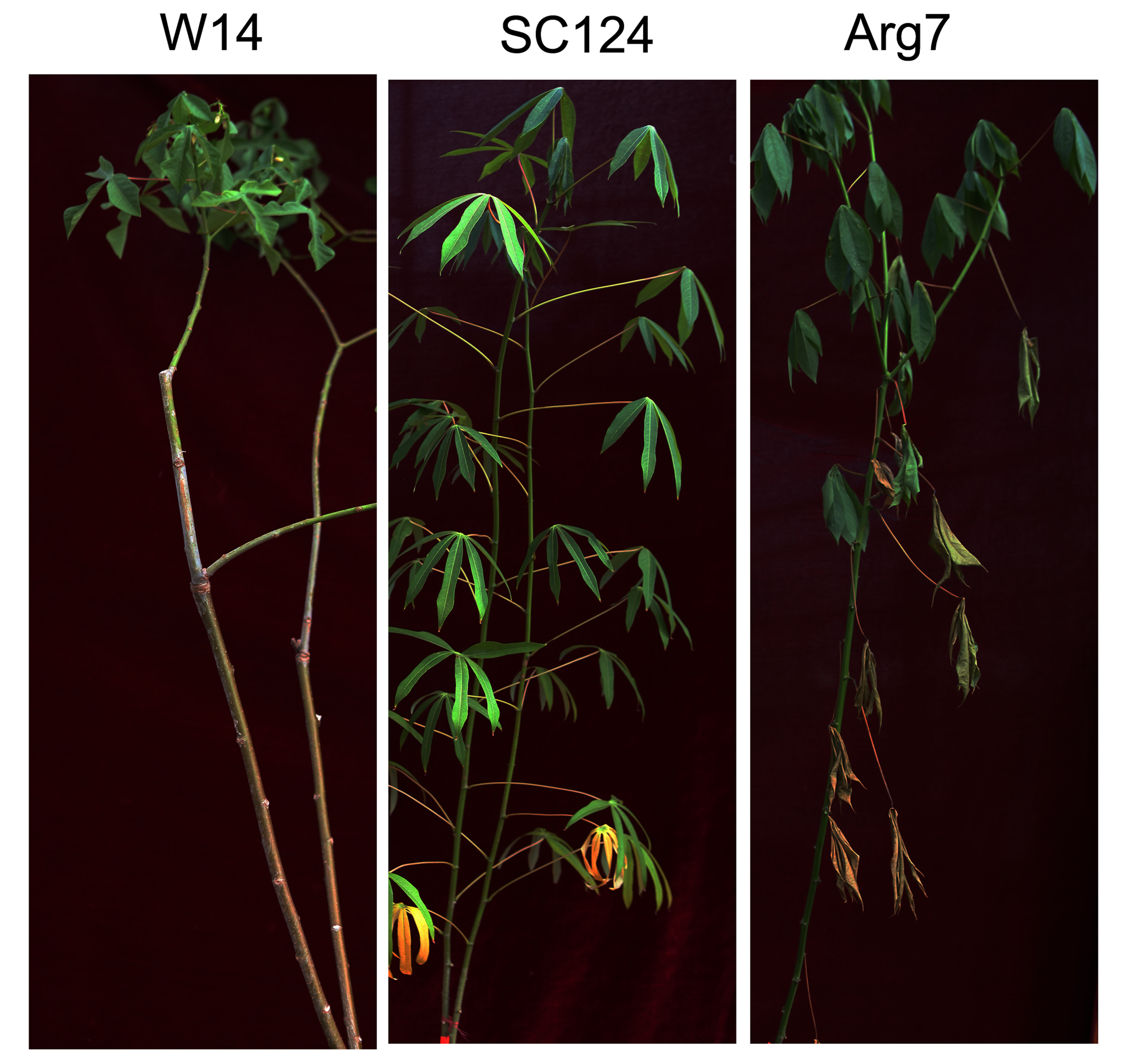


**Supplementary Figure S7.**  **Photos of different varieties of cassava after 12 days drought treatment.**
